# THE INFLUENCE OF THE COVID-19 PANDEMIC ON DOMESTIC ELDER ABUSE IN JAPAN

**DOI:** 10.1093/geroni/igae098.1849

**Published:** 2024-12-31

**Authors:** Tadashi Wada, Hitoshi Suda, Tamiko Uehara, Kana Sato

**Affiliations:** Hidamari Homecare Clinic, Chiba, Chiba, Japan; Seitoku University, Matsudo-city, Chiba, Japan; Matsudo City Medical Association, Matsudo City, Chiba, Japan; Teikyo University of Science & Technology, Adachi-ku, Tokyo, Japan

## Abstract

We are very interested in the influence of COVID-19 pandemic on elder abuse, especially its effects on the incidence. We investigated its incidence per population of validated domestic elder abuse cases in Matsudo City, Chiba, Japan, and in all over Japan. The Act of the Prevention of Elder Abuse, Support for Caregivers of Elderly Persons and Other Related Matters was enforced in 2004. We have the statistics of numbers of elder abuse cases validated according to the definition of elder abuse described in the act of each annual year in Matsudo City and Japan from 2004 to 2022. The numbers of validated domestic elder abuse cases per 100,000 people between annual 2004 and 2019 were 81.8 in Matsudo City and 49.8 in Japan. The numbers per 100,000 people between annual 2020 and 2022 were in 84.1 in Matsudo City and 46.4 in Japan. Therefore, the number of domestic elder abuse cases may be slightly increased under the COVID-19 pandemic in Matsudo City, on the contrary, it may be slightly decreased in Japan. There are many reports that the COVID-19 pandemic had negative effects on dynamics of family members and increased domestic abuses, however, we have often experienced to see positive effects of the COVID-19 pandemic on family members in home-care settings. I would like to discuss about the influence of the COVID-19 pandemic on the number of domestic elder abuse cases in Matsudo City and in Japan, and its positive and negative effects on family dynamics at home.

